# Bronchopulmonary dysplasia and neurodevelopmental outcome in extremely preterm neonates

**DOI:** 10.1007/s00431-013-2016-5

**Published:** 2013-05-05

**Authors:** J. K. Trittmann, L. D. Nelin, M. A. Klebanoff

**Affiliations:** Ohio Perinatal Research Network, Center for Perinatal Research, The Research Institute at Nationwide Children’s Hospital, Department of Pediatrics, The Ohio State University College of Medicine, 700 Children’s Drive, Research Building III, WB 5245, Columbus, OH 43205 USA

**Keywords:** Mechanical ventilation, Supplemental oxygen, Very low birth weight, Intraventricular hemorrhage, Necrotizing enterocolitis

## Abstract

We tested the hypothesis that the use of supplemental oxygen (sO_2_) at discharge from the NICU in extremely preterm neonates is associated with a greater risk of neurodevelopmental impairment (NDI) at 18 months corrected gestational age (CGA) than the risk of NDI of those neonates discharged in room air. Four hundred twenty-four charts were retrospectively reviewed from infants born at <27 weeks and transferred to Nationwide Children’s Hospital from December 1, 2004 to June 14, 2010. Use of sO_2_ was evaluated on day of life (dol) 28, at 36 weeks post-menstrual age (PMA), and at discharge. Logistic regression was used to identify postnatal risk factors associated with sO_2_ at discharge and NDI. At dol 28, 96 % of surviving patients received sO_2_, and therefore had bronchopulmonary dysplasia (BPD) by definition from a National Institutes of Child Health and Human Development workshop. At 36 weeks PMA, 89 % continued on sO_2_ (moderate/severe BPD), and at discharge, 74 % continued on sO_2_. When factors associated with NDI were examined, the need for mechanical ventilation ≥28 days (adjOR = 3.21, *p* = 0.01), grade III–IV intraventricular hemorrhage (IVH) (adjOR = 4.61, *p* < 0.01), and discharge at >43 weeks PMA (adjOR = 2.12, *p* = 0.04) were the strongest predictors of NDI at 18 months CGA. There was no difference in Bayley Scales of Infant Development, third edition composite scores between patients with no/mild BPD and patients with moderate/severe BPD (cognitive *p* = 0.60, communication *p* = 0.53, motor *p* = 0.19) or those scores between patients on and off oxygen at discharge (cognitive *p* = 0.58, communication *p* = 0.70, motor *p* = 0.62). *Conclusions*: The need for sO_2_ at discharge is not associated with an increased risk of NDI in these patients. The strongest predictors of poor neurodevelopmental outcome in this population were prolonged positive pressure support, grade III–IV IVH, and discharge at >43 weeks PMA.

## Introduction

Bronchopulmonary dysplasia (BPD) is the most common chronic lung disease affecting preterm infants, and the incidence of BPD is inversely related to gestational age [37]. In 1967, Northway first described BPD as a requirement for supplemental oxygen (sO_2_) at day of life (dol) 28 with chest X-ray findings demonstrating fibrosis and collapse surrounded by areas of marked hyperinflation [26]. Over the decades, a “new” BPD has emerged that is characterized by fewer and larger alveoli, as well as decreased pulmonary microvasculature development [11, 18, 20, 35]. The emergence of “new” BPD and the improved survival of patients born at extremely early gestational ages led to a new definition of BPD from a National Institutes of Child Health and Human Development (NICHD) workshop [18]. Using this definition and the inverse relationship to gestational age, the incidence of BPD in the extremely preterm infant is very high. For example, Farstad et al. recently reported an incidence of 86 % in a Norwegian cohort of patients born between 22 and 27 completed weeks of gestation [12]. Given the high incidence of BPD in this population, we examined the use of sO_2_ at discharge to determine incidence and factors associated with sO_2_ use at discharge. Since BPD has been associated with poor neurodevelopmental outcome [3, 14, 17, 27–29, 31–34, 38], we tested the hypothesis that the use of sO_2_ at discharge from the NICU in extremely preterm neonates is associated with a greater risk of neurodevelopmental impairment (NDI) at 18 months corrected gestational age (CGA).

## Methods

This study was approved by the Institutional Review Board of the Nationwide Children’s Hospital (NCH). This is a retrospective observational study. Charts were reviewed and data obtained from all 424 patients who were born at <27 weeks gestation and transferred to the NCH NICU between December 1, 2004 and June 14, 2010. Only patients who survived, were not transferred, and had available data at 28 days were included in the study (345 patients). The NCH NICU is an all referral (i.e., no inborn patients) level IV [1] center in Columbus, Ohio, USA, which receives referrals from 22 hospitals in Central Ohio. The NICU Small Baby Pod at NCH is a dedicated space and nursing staff which allows for an interdisciplinary, standardized approach to the care of the extremely preterm neonate born at <27 completed weeks gestational age [24].

NICU data were abstracted from charts by trained personnel and entered into the Small Baby Database, which contains basic demographic information and details of the hospital course for each patient admitted to the Small Baby Pod at NCH. Gestational age was determined by prenatal ultrasound when available and confirmed by Ballard Score. Since NCH is an all referral hospital, maternal data were limited. For example, the presence of chorioamnionitis was not reliably available on all patients. Oxygen status was assessed at dol 28, 36 weeks post-menstrual age (PMA), and at discharge. The attending physician prescribed the sO_2_ for these patients. At the time this study was conducted, there was not a policy to include an oxygen challenge test at 36 weeks and supplemental oxygen was as prescribed by the attending neonatologist. BPD was defined as a need for sO_2_ at dol 28 and then stratified based on the use of sO_2_ at 36 weeks PMA as per the definition from a NICHD workshop [18], where mild disease is defined as no sO_2_ at 36 weeks PMA, moderate BPD is defined as an sO_2_ requirement of <30 % at 36 weeks PMA, and severe BPD is defined as an sO_2_ requirement of >30 % and/or positive pressure support at 36 weeks PMA. Comorbidities were assessed, including necrotizing enterocolitis (NEC) and intraventricular hemorrhage (IVH). Short-term outcome measurements included sO_2_ at discharge and hospital length of stay. Longer term outcomes included 18-month corrected composite scores on the Bayley Scales of Infant Development, third edition (BSID-III) assessment. Patients without BPD were evaluated at routine follow-up visits in the neonatology outpatient clinic and patients with BPD were evaluated at the Comprehensive Center for BPD at NCH [30]. NDI was defined as any Bayley composite score (cognitive, communication, or motor) <80 or cerebral palsy [19, 22, 39].

## Statistical analysis

Patients who were discharged to home (i.e., survived and not transferred to another institution) were divided into two groups. The first group consisted of those patients who did not require sO_2_ at discharge. The second group consisted of those patients who required sO_2_ at discharge. Continuous variables, including BSID-III scores, were compared between groups using Student’s *t* test, and categorical variables, including NDI, were compared by *χ* ^2^ analysis. Logistic regression was used to model those factors potentially associated with sO_2_ at discharge and NDI. Clinical characteristics were chosen based on a priori interest and included ≤24 weeks gestational age, birth weight ≤700 g, Apgar score at 5 min ≤3, admission dol ≥7, CPAP ≥28 days, mechanical ventilation ≥28 days, PDA ligation, surgical NEC, grade III–IV IVH, discharge >43 weeks PMA, moderate–severe BPD, and sO_2_ at discharge. Unadjusted and adjusted odds ratios for NDI were also calculated for these same clinical characteristics. All analyses were conducted with STATA 12 statistical software (StataCorp. 2011. Stata Statistical Software: Release 12. College Station, TX: StataCorp LP). Boxplots were created using GraphPad Prism version 6.00 for Windows (GraphPad Software, La Jolla California USA, www.graphpad.com). A two-tailed *p* value of <0.05 was considered statistically significant.

## Results

**Fig. 1:**
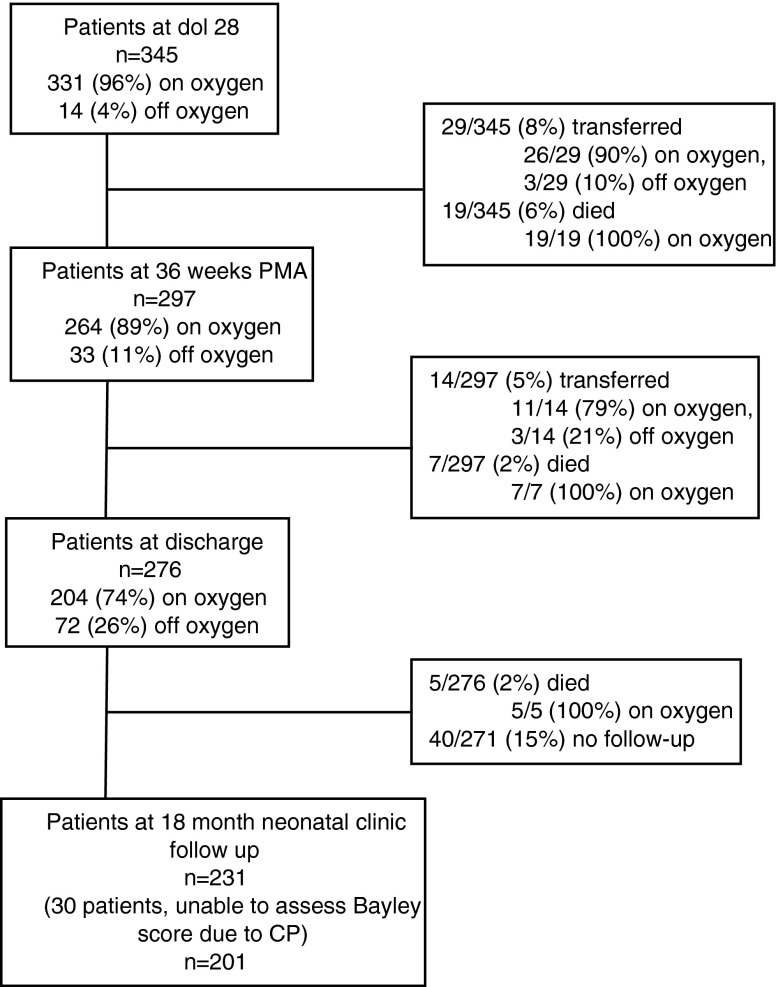
Patients admitted between December 1, 2004 and June 14, 2010. Three hundred forty-five patients born at <27 weeks gestation were eligible for this study on day of life (dol) 28. At discharge, there remained 276 patients of which 2 % died and 15 % did not return for follow-up at the neonatal clinic. At 18 months corrected gestational age, 30 patients had cerebral palsy (*CP*) and 201 patients had Bayley scores available for analysis

Of the 424 patients admitted to NCH during the time of this study, 16 (4 %) transferred prior to dol 28, 55 (13 %) died prior to dol 28, and 8 (2 %) did not have available data at dol 28, such that there were 345 eligible patients at dol 28 (Fig. 1). Figure 1 also demonstrates the number of eligible patients at 36 weeks PMA, discharge to home, and at 18-month follow-up. It is of interest to note that for this highly vulnerable patient group, the overall mortality rate during the initial NICU stay was 20 % (86/424), and most of the mortality occurred before dol 28, resulting in a mortality rate between dol 28 and discharge of 7 %. Furthermore, there was no mortality in patients during their initial NICU stay that were successfully weaned off of sO_2_ from dol 28 until discharge.

Table 1 summarizes sO_2_ use at dol 28, 36 weeks PMA, and discharge in the extremely preterm infant stratified by gestational age. For the entire group, 96 % of the patients received sO_2_ at dol 28, 89 % continued to receive sO_2_ at 36 weeks PMA, and 74 % of the patients continued to receive sO_2_ at discharge. Only 4 % of the patients did not require sO_2_ at dol 28 and therefore, by NIH definition, did not have BPD [18]. Even in this extremely preterm population, the use of sO_2_ at any of the measured time points tended to be greater as gestational age of the patient at birth decreased (*p* < 0.01).

**Table 1:** Supplemental oxygen (sO_2_) use at day of life (dol) 28, 36 weeks post-menstrual age (PMA), and at discharge for patients according to gestational age at birth

Gestational age (weeks)	sO_2_ at 28 dolª	sO_2_ at 36 weeks PMAª	sO_2_ at dischargeª
22–23	100 % (55/55)	91 % (42/46)	86 % (37/43)
24	98 % (95/97)	90 % (76/84)	75 % (56/75)
25	95 % (98/103)	91 % (82/90)	74 % (64/86)
26	92 % (83/90)	83 % (64/77)	65 % (47/72)
Total	96 % (331/345)	89 % (264/297)	74 % (204/276)

ªDenominator represents all infants alive and in the NICU at that point in time. Percent (ratio) determined for dichotomous variables.

Table 2 summarizes patient characteristics, selected comorbidities, and NDI for this population. Those patients who went home on sO_2_ were of lower gestational age (*p* = 0.02) and weighed at birth an average of 50 g less than patients discharged on room air (*p* = 0.03). Patients discharged on sO_2_ had significantly (*p* < 0.01) more days on mechanical ventilation (58 ± 51 days) than did patients discharged on room air (24 ± 21 days). Patients discharged on sO_2_ had a PDA ligation more often (52 vs. 36 %, *p* = 0.02) and had less surgical NEC (8 vs. 28 %, *p* < 0.01) than did patients discharged on room air. NDI was similar between those discharged on room air and those discharged on sO_2_ (51 vs. 47 %, *p* = 0.67).

**Table 2:** Supplemental oxygen (sO_2_) use at discharge according to patient characteristics

Characteristics	Total (*n* = 276)	No sO_2_ at discharge (*n* = 72)	sO_2_ at discharge (*n* = 204 )	*p* value
Gestational age (weeks)	24.7 ± 1.1	24.9 ± 1.0	24.6 ± 1.1	0.02
Birth weight (grams)	723 ± 166	760 ± 159	710 ± 167	0.03
5-min Apgar score	6 ± 2	6 ± 2	6 ± 2	0.17
Age on admission (dol)	11 ± 12	11 ± 12	10 ± 12	0.44
CPAP (days)	35 ± 19	31 ± 16	36 ± 20	0.06
Mechanical ventilation (days)	49 ± 47	24 ± 21	58 ± 51	<0.01
PDA ligation	48 % (133/276)	36 % (26/72)	52 % (107/204)	0.02
Surgical NEC	13 % (36/276)	28 % (20/72)	8 % (16/204)	<0.01
Grade III–IV IVH	21 % (59/275)	18 % (13/72)	23 % (46/203)	0.41
Length of stay (days)	146 ± 61	136 ± 54	150 ± 63	0.08
Length of stay (PMA, weeks)	47 ± 9	46 ± 7	47 ± 9	0.19
Neurodevelopmental Impairmentª	50 % (115/231)	47 % (26/55)	51 % (89/176)	0.67

ªAny Bayley composite score (cognitive, communication, or motor) <80 or cerebral palsy

In the adjusted analysis of sO_2_ at discharge, patients receiving CPAP for ≥28 days were more likely to go home on oxygen (adjOR = 2.9, 95 % CI 1.4–6.2, *p* < 0.01). Similarly, patients receiving ≥28 days of mechanical ventilation were more likely to receive sO_2_ at discharge (adjOR = 7.7, 95 % CI 2.9–20.5, *p* < 0.01). We also found that there was a significantly (*p* < 0.01) greater proportion of patients in the discharged home on room air group that were ventilated for ≤14 days (45 %) than in those discharged home on sO_2_ (20 %). Patients with surgical NEC were less likely to receive sO_2_ at discharge (adjOR = 0.11, 95 % CI 0.04–0.30, *p* < 0.01). None of the other factors in Table 2 were significantly associated with sO_2_ at discharge in the logistic regression model.

In order to further explore the relationship between surgical NEC and sO_2_ at discharge, we analyzed the characteristics of these 36 patients. The mortality rate (after dol 28) for surgical NEC was 2/36 (6 %). Those surgical NEC patients who were discharged on sO_2_ had a longer (*p* = 0.02) duration of mechanical ventilation (58 ± 31 days) than did those surgical NEC patients discharged on room air (35 ± 27 days). However, patients with surgical NEC who went home on sO_2_ had a significantly shorter length of stay (surgical NEC home on sO_2_ 144 ± 28 days vs. surgical NEC home on room air 188 ± 69 days, *p* = 0.02) and their PMA at time of discharge was less (46 ± 4 weeks vs. 53 ± 10 weeks, *p* < 0.01) than those patients with surgical NEC who went home on room air.

**Fig. 2:**
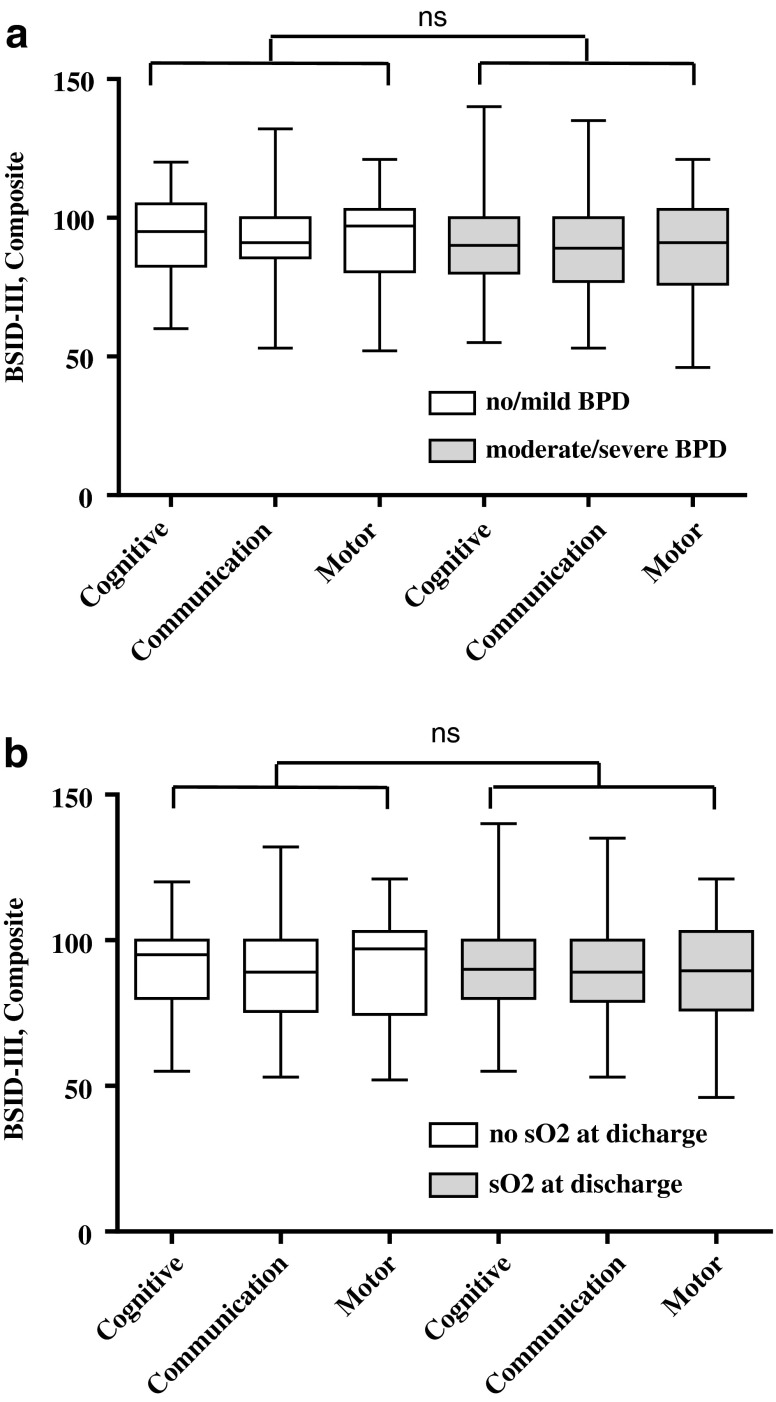
BSID-III composite scores (cognitive, communication, and motor) of neonates born at <27weeks gestation (*n* = 201). Boxplots represent median and interquartile range. **a** Boxplots comparing no/mild BPD to those neonates with moderate/severe BPD. Student’s *t* test for cognitive score (*p* = 0.60), communication score (*p* = 0.53), and motor score (*p* = 0.19). There was no significant difference (ns) between the no/mild BPD and moderate/severe BPD groups. **b** Boxplots comparing no supplemental oxygen (*sO*_*2*_) at discharge to those neonates receiving sO_2_ at discharge. Student's *t* test for cognitive score (*p* = 0.58), communication score (*p* = 0.70), and motor score (*p* = 0.62). There was no significant difference (ns) between the no sO_2_ at discharge and sO_2_ at discharge groups

Of the 276 patients who were discharged alive, 5 subsequently died and 231 (85 % of survivors) presented for an 18-month Bayley exam at their routine neonatal clinic follow-up visit. Cerebral palsy was diagnosed in 30/231 (13 %) of those patients discharged alive who presented for follow-up (Fig. 1). Given that Bayley scores were not assigned for patients with cerebral palsy, Bayley scores were available for 201 patients. Motor scores were not available for three patients and communication scores were not available for two patients, due to inability to perform the test. For the corrected cognitive scores, 54/201 (27 %) were <80; for the corrected communication scores, 65/199 (33 %) were <80; and for the corrected motor scores, 62/198 (31 %) were <80. There were no significant differences in Bayley scores between those patients with no/mild BPD and moderate/severe BPD (Fig. 2a). Similarly, there were no significant differences in Bayley scores off and on sO_2_ at discharge (Fig. 2b).

Table 3 summarizes the unadjusted and adjusted odds ratios for NDI. Unadjusted odds ratios were significant for predicting NDI for those patients born at ≤24 weeks gestational age, birth weight ≤700 g, mechanical ventilation ≥28 days, surgical NEC, grade III–IV IVH, and discharge at >43 weeks PMA. After adjustment for all the factors in Table 3, only ventilation for ≥28 days, grade III–IV IVH, and discharge at >43 weeks PMA remained significantly associated with NDI. Oxygen at discharge was not predictive of NDI (adjOR 0.67, *p* = 0.18).

**Table 3:** Unadjusted and adjusted odds ratios for neurodevelopmental impairment (NDI) by patient characteristic

	Unadjusted			Adjusted		
	OR	CI	*p* value	OR	CI	*p* value
≤24weeks	2.14	(1.26–3.63)	<0.01	1.88	(0.90–3.91)	0.09
≤700 g	2.46	(1.45–4.17)	<0.01	1.70	(0.82–3.54)	0.15
Apgar 5 min ≤3	1.85	(0.86–4.0)	0.12	1.85	(0.75–4.59)	0.18
Admit dol ≥7	0.82	(0.48–1.39)	0.47	0.92	(0.46–1.82)	0.80
CPAP ≥28 days	0.96	(0.57–1.64)	0.89	1.42	(0.71–2.86)	0.32
Ventilation ≥28 days	4.08	(2.28–7.32)	<0.01	3.21	(1.34–7.70)	0.01
PDA ligation	0.89	(0.53–1.48)	0.64	0.63	(0.33–1.23)	0.17
Surgical NEC	3.68	(1.51–9.01)	<0.01	2.54	(0.83–7.78)	0.10
Grade III–IV IVH	4.90	(2.36–10.18)	<0.01	4.61	(1.8–11.8)	<0.01
Discharge at >43 weeks PMA	3.22	(1.86–5.59)	<0.01	2.12	(1.04–4.32)	0.04
Moderate–severe BPD	1.49	(0.61–3.63)	0.38	0.59	(0.16–2.16)	0.43
Oxygen at discharge	1.14	(0.62–2.09)	0.67	0.52	(0.20–1.36)	0.18

NDI defined as any Bayley composite score (cognitive, communication, or motor) <80 or CP

## Discussion

In this cohort of infants born at <27 weeks gestational age, transferred to an all referral NICU, and surviving to dol 28, the majority of surviving patients had BPD as defined by a sO_2_ requirement on dol 28. Our data are consistent with a recent study of a national cohort by The Norwegian Extreme Prematurity Study Group, which found that 86 % of infants born between 22 and 27 weeks gestation had BPD and that 80 % of infants born at 22 to 25 completed weeks gestation had moderate-to-severe BPD [12]. In our retrospective cohort of infants referred to a level IV Children’s Hospital NICU, 89 % had moderate-to-severe BPD. Unlike other reported cohorts of extremely preterm infants, our rate of moderate-to-severe BPD is likely influenced by the fact that all of our patients are outborn. Outborn patients are referred from several level III NICUs and include the sickest extremely preterm infants from throughout our region, many of whom are referred when they remain on supplemental oxygen. The NICHD Neonatal Research Network recently reported that 43 % of inborn neonates <29 weeks gestation had moderate-to-severe BPD [36]. Given that the incidence of BPD is inversely related to gestational age at birth [11, 12, 18, 20, 26, 35, 37], differences in the gestational age of included infants likely contributes to variation in published rates of moderate–severe BPD.

In our extremely preterm infants who survived to discharge, the vast majority (74 %) were discharged home on sO_2_. As expected, those patients requiring sO_2_ at discharge were born earlier and weighed less at birth than those patients who did not require sO_2_ at discharge. Patients who went home on sO_2_ had a longer duration of mechanical ventilation. We found that the use of mechanical ventilation for ≥28 days is the strongest predictor of need for sO_2_ at time of discharge in these patients. In our patient cohort, there was no difference in length of stay between patients who were discharged on sO_2_ and those discharged on room air. Therefore, it does not appear that patients were being sent home on sO_2_ in order to hasten discharge from the NICU.

We were surprised to find that patients with surgical NEC were less likely to receive sO_2_ at discharge. Therefore, we examined patient characteristics that might contribute to this finding. Patients diagnosed with surgical NEC and discharged home on room air had a significantly longer length of stay, as well as greater PMA at discharge, than those surgical NEC patients who were discharged on sO_2_. This suggests that in the subgroup of patients with surgical NEC, those discharged on room air remained in hospital longer, and during this longer hospital stay (an average of ~7 weeks) were able to wean from sO_2_.

The BSID-III was used to assess neurodevelopmental outcome. Recently, it has been suggested that the BSID-III underestimates the severity of disability as compared to the BSID-II [2, 19]. Therefore, we followed published recommendations and used a composite score of <80 on any of the three categories (cognitive, communication, or motor) or cerebral palsy to denote NDI [22]. Our rates of NDI were similar to a recent report by Moore et al. in the same gestational age group [22]. There was no significant difference in Bayley scores between those patients with no/mild BPD vs. moderate/severe BPD or those patients discharged on sO_2_ vs. room air for any of the three categories. Thus, BPD severity or need for sO_2_ at discharge in this cohort of patients does not adversely affect neurodevelopmental outcome. Although there is literature to suggest BPD predicts worsening NDI, many of these patients were evaluated prior to 2000 [3, 9, 25]. Other studies have suggested that BPD may not be an independent risk factor of poor neurodevelopmental outcome [13]. Our study population was evaluated between years 2004 and 2010, so it may be that more recently there has been an improvement in neurodevelopmental outcome in patients with BPD. Consistent with this concept are at least three recent studies. Hintz et al. found worsening rates of BPD while NDI remained relatively constant over time [16]. The EPICure studies found that in the most recent patient cohort compared to earlier cohorts there were more neonates born at less than 27 weeks surviving without disability [21]. Finally, a recent systematic review of NDI rates in extremely preterm infants found a significant decrease in rates of cerebral palsy based on increasing year of birth [6].

At NCH, we developed an innovative program directed at this population of extremely preterm infants, the Small Baby Program, that started on December 1, 2004 [23]. The adoption of this program which is based on a standardized approach to the care of these infants led to an improvement in hospital outcomes [23]. Furthermore, we found improved survival and neurodevelopmental outcomes at 18–24 months compared to similar cohorts of patients [23]. We also found at about the same time that the care given to patients with established moderate-to-severe BPD was highly variable and often lacked a neurodevelopmental focus during the initial NICU stay. Therefore, we implemented a standardized inpatient and outpatient program for these patients focused on developmental needs [29]. These programs may have impacted the favorable outcome that BPD patients in this study had who were discharged home on supplemental oxygen.

There are some notable limitations of our study. The population admitted to an all referral children’s hospital may not be representative of all extremely preterm neonates. For example, in our referral area, most extremely preterm infants are born in delivery hospitals with a level III NICU and are only transferred if there is need for surgical or subspecialty care. It should also be kept in mind that the extremely preterm neonates included in our study have survived both the delivery room and transport to our institution. We are an all referral center and therefore have limited ability to extract maternal data, as well as resuscitation data, from outside institutions for research purposes. Also, our study included information on 18-month Bayley scores; a recent systematic review found that higher rates of attrition were associated with worse neurodevelopmental outcome [15]. Although only 85 % of our patients returned for follow-up, this is a relatively high follow-up rate for the USA [6] and improved follow-up would be expected to improve overall NDI. Lastly, at the time of this study, there was no policy in place to objectively measure the need for oxygen. However, since then, we have implemented a 36-week oxygen challenge test and are following the data to determine if there is a decrease in the incidence of BPD and the use of supplemental oxygen at discharge.

The current definition of BPD depends on the need for and the amount of sO_2_ given to a patient [4–8]. Oxygen weaning varies by clinical practice among neonatologists and among centers. This may be responsible for some of the wide variation in BPD incidence among NICUs [10]. The currently used criteria for defining BPD results in the vast majority of patients born at <27 weeks having moderate-to-severe BPD. This is consistent with recent studies from the USA [6, 36], Norway [12], and Sweden [11] in a similar gestational age cohort. Further studies are needed to develop BPD phenotypes more specific to the extremely preterm infant that could be used to more effectively and efficiently develop preventative strategies and predict neonatal outcomes.

In summary, we report for the first time that the need for sO_2_ at discharge is not an independent predictor of poor neurodevelopmental outcome in patients born before 27 completed weeks gestational age. When examining variables that might predict discharge on supplemental oxygen, not unexpectedly long-term mechanical ventilation was the most predictive of discharge home on sO_2_ in this population. These findings underscore the need to develop strategies and therapies that decrease the length of time that mechanical ventilation is required to improve pulmonary outcomes in these patients. Given that the diagnosis of BPD is based on supplemental oxygen requirement, our data suggest that in the extremely preterm infant BPD as currently defined is found in nearly all patients. Therefore, we suggest that for the neonate born at the edge of viability there is a need for developing phenotypic criteria for BPD in order to better characterize disease severity and predict long-term outcomes. The data from this relatively large and recent cohort of extremely preterm infants demonstrate that discharge home on supplemental oxygen does not in and of itself translate to a poor neurodevelopmental outcome.

## Abbreviations

BPD: Bronchopulmonary dysplasia
dol: Day of life
IVH: Intraventricular hemorrhage
NEC: Necrotizing enterocolitis
NCH: Nationwide Children’s Hospital
PMA: Post-menstrual age
sO_2_: Supplemental oxygen
